# Transcriptome Profiling of Lung Innate Immune Responses Potentially Associated With the Pathogenesis of *Acinetobacter baumannii* Acute Lethal Pneumonia

**DOI:** 10.3389/fimmu.2020.00708

**Published:** 2020-04-22

**Authors:** Xi Zeng, Hao Gu, Liusheng Peng, Yao Yang, Ning Wang, Yun Shi, Quanming Zou

**Affiliations:** ^1^Department of Microbiology and Biochemical Pharmacy, National Engineering Research Center of Immunological Products, College of Pharmacy, Army Medical University, Chongqing, China; ^2^Institute of Biopharmaceutical Research, West China Hospital, Sichuan University, Chengdu, China; ^3^Department of Clinical Laboratory, 971st Hospital of People's Liberation Army, Qingdao, China; ^4^Institute of Materia Medica, College of Pharmacy, Army Medical University, Chongqing, China

**Keywords:** *Acinetobacter baumannii*, acute pneumonia, RNA-seq, innate immune response, pathogenesis

## Abstract

*Acinetobacter baumannii* is one of the dominating causes of nosocomial pneumonia, however, very little is known about the host immune response associated with pathogenesis of *A. baumannii* infection. Here, we used a hypervirulent *A. baumannii* to establish an acute lethal pneumonia, supported by high bacterial burdens, severe inflammatory cells infiltration and lung damage. The lung transcriptome changes in response to *A. baumannii* lethal pneumonia were detected by RNA sequencing. The results showed that 6,288 host genes changed expression, with 3,313 upregulated genes and 2,975 downregulated genes. Gene Ontology and Kyoto Encyclopedia of Genes and Genomes pathway analysis revealed that genes related to TNF, cytokine-cytokine receptor interaction, Toll-like receptor, NOD-like receptor, NF-κB, Jak-STAT, HIF-1 signaling pathways, apoptosis, and phagosome were significantly upregulated. Whereas, genes associated with PI3K-AKT signaling pathway, glycolysis/gluconeogenesis, amino acid and fatty acid metabolism were downregulated. Immune cell typing highlighted the inflammatory response of innate immune cells headed by neutrophils. The reliability of RNA sequencing results were verified with selected differentially expressed genes by real-time PCR. This work provides an insight into the pathogenesis of lethal *A. baumannii* lung infection.

## Introduction

Multi-drug resistance *Acinetobacter baumannii* has been list as top threat to public health by World Health Organization, because of the severe drug resistance and the serious infections. It can cause pneumonia, blood infection, urinary tract infection, wound infection and meningitis. The most disturbing infection caused by *A. baumannii* is nosocomial pneumonia or ventilator-associated pneumonia (1, 2), which can results in 40–70% mortality (3, 4). However, the host immune response associated with pathogenesis of *A. baumannii* pneumonia is less elucidated.

Previous works showed that *A. baumannii* usually induces an acute infection, in which innate immune system including neutrophils and macrophages play a dominant role in host defense (5–7). However, most conclusions are drawn from the non-lethal model using low-virulent strains, which induce a self-limiting pneumonia (7, 8). In this circumstance, host immune responses exhibit a transitional inflammation in the lung and eventually control the bacterial infection. Recently, more hypervirulent strains of *A. baumannii* are isolated and used to establish lethal mice model and more researchers explored the governing host factors associated with *A. baumannii* lethality (9, 10). It has been reported that *A. baumannii* infection induced the production of proinflammatory cytokines TNF-α, type I IFN, and IL-1β, which mediated cell death and lung pathology (11–14). The induction of potent inflammatory immune response contributed to the mortality caused by *A. baumannii*. These indicated that the innate immune response is a double-edge sword and the exaggerated inflammatory responses are also detrimental to host tissue (15). More comprehensive description of innate immune response related to pathogenesis during acute *A. baumannii* pneumonia is needed.

Here, we studied the lung transcriptome changes during *A. baumannii*-induced acute lethal pneumonia in mice. The results showed that 6,288 host genes have changed their expression following the infection. We further analyzed the differentially expressed genes by matching them in the GO and KEGG pathways. It will help us to understand the pathogenesis of *A. baumannii* lung infection, and to find novel therapeutic targets.

## Materials and Methods

### Mice and Bacteria Strain

Wild-type C57BL/6 mice were purchased from Beijing HFK Bioscience Limited Company (Beijing, China). Rag1 gene knockout mice (Rag1^−/−^, B6.129S7-Rag1tm1Mom/J) were purchased from Model Animal Research Center of Nanjing University. All mice were female, housed under specific pathogen free conditions, infected at 6–8 weeks of age. All the animal experiments were approved by the Animal Ethical and Experimental Committee of the Amy Medical University. *A. baumannii* strain LAC-4 was kindly provided by Prof. Chen.

### Mouse Pneumonia Model

WT and Rag1^−/−^ mice were infected with *A. baumannii* as previously described (16). Briefly, Mice were intraperitoneally anesthetized with pentobarbital sodium (62.5 mg/kg) and then non-invasive intratracheally inoculated with different dose (2.5 × 10^6^ CFU, 5 × 10^6^ CFU, and 2 × 10^7^ CFU) of *A. baumannii* in 20 μl PBS. The survival of mice was observed for 7 days post infection. Infected WT mice were sacrificed at 0, 24, 48, 72 h post infection (hpi), the lungs were collected to determined histopathology, the blood and lung homogenate were diluted and cultured on plates to determined bacterial burdens. The lungs from mice infected with 2 × 10^7^ CFU were collected at 24 hpi to determined cytokine/chemokine levels. Rag1^−/−^ mice were infected with 2 × 10^7^ CFU of *A. baumannii* and then sacrificed at 24 hpi, the lungs were processed to do RNA-seq.

### Histopathology Analysis

Lungs were fixed in 4% paraformaldehyde and paraffin-embedded. Tissue sections were stained with hematoxylin and eosin and examined under light microscopy.

### RNA Extraction, Library Construction and Sequencing

Total RNAs from lung samples of Rag1^−/−^ mice were extracted using TRIzol (Invitrogen). And then DNA digestion was carried out by DNaseI. RNA quality was evaluated by examining A260/A280 with Nanodrop^TM^ One spectrophotometer (Thermo Fisher Scientific Inc.). RNA Integrity was determined by 1.5% agarose gel electrophoresis. RNAs were quantified by Qubit3.0 with Qubit^TM^ RNA Broad Range Assay kit (Life Technologies). Finally, 2 μg total RNAs were used to prepare KC-Total RNA-seq Library Prep Kit for Illumina® (Catalog NLR086-01, Wuhan Seqhealth Co., Ltd. China). PCR products corresponding to 200–500 bps were enriched, quantified and sequenced on Hiseq X 10 sequencer (Illumina).

### Analysis of RNA-Seq Data

Raw data quality control was performed using FastQC software. Raw data clean was done with Trimmomatic software. The clean data were mapped to the mouse genome GRCm38 with STAR software. Mapped reads distribution, coverage uniformity, and strand specificity were evaluated by RSeQC. The reads count for each gene were calculated using FeatureCounts, and expressed as RPKM (reads per kilobase per million reads). Differentially expressed genes (DEGs) were identified using edgeR. The absolute value of logFC >1 and *p*-value < 0.05 was taken as the standard, indicating that the gene was differentially expressed. Kobas software was applied to Gene Ontology (GO) and Kyoto Encyclopedia of Genes and Genomes (KEGG) enrichment. was conducted with. Enrichr (https://amp.pharm.mssm.edu/Enrichr/) was also used to conduct GO classification. Hierarchical clustering and heatmaps were drawn with pheatmap R package and MA-plot was drawn with edgeR package.

### Immune Cell Typing

ICEPOP (Immune CEll POPulation, interactive web site: https://vdynamics.shinyapps.io/icepop/, Python package: https://github.com/ewijaya/icepop) was used to estimate immune responses in individual immune cell types. The relative gene responses were scored for each cell type by using the data from DEGs (fold-change >2.0), and the data from public databases including ImmGen (http://www.immgen.org/) and IRIS (http://share.gene.com/share/clark.iris.2004/iris/iris.html), which contained gene expression profiles of various immune cells. In these databases, different immune cell subtypes were grouped into 10 immune cell types.

### Real-Time PCR

Total RNA was extracted using RNAiso Plus (Takara) and reverse transcribed to cDNA using PrimeScript^TM^ RT reagent Kit (Takara). The mRNA expression was detected using SYBR green Premix (Takara) with specific primers described previously (16). Real-time PCR reactions were performed on CFX96 (Bio-Rad). The expression levels of genes were determined relative to uninfected mice controls. Gene expression was normalized to the Ct values for β-actin using the formula 2^Δ^ΔCT.

### Statistical Analysis

Statistical analysis was analyzed using GraphPad Prism software (version 6.01). Student's *t*-test was applied for comparison of two data sets, the data were presented as means ± SEM. *P* < 0.05 was considered statistically significant.

## Results

### A Lethal Pneumonia Induced by *A. baumannii*

**Figure 1:**
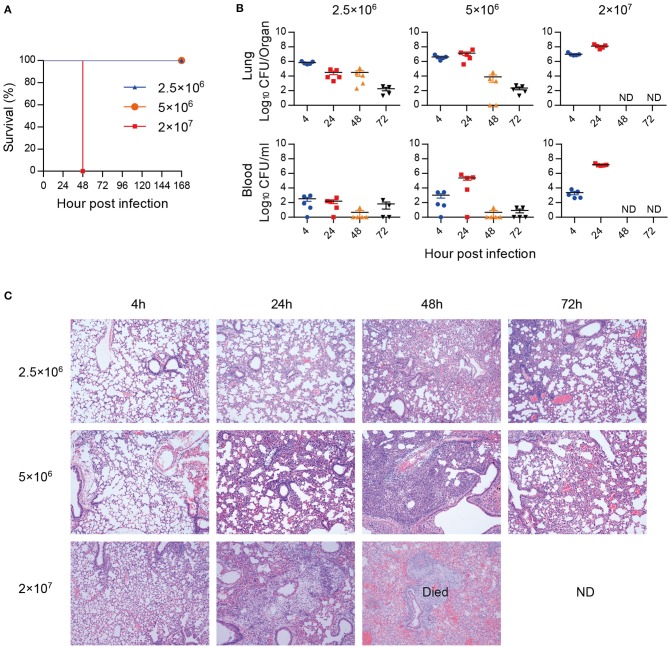
*A. baumannii*-induced lethal pneumonia. Groups of C57BL/6 mice (*n* = 4–5) were intratracheally inoculated with 2.5 × 10^6^, 5 × 10^6^, and 2 × 10^7^ CFU of *A. baumannii* LAC-4. **(A)** The survival rates were monitored daily for 7 days. **(B,C)** Bacterial burdens **(B)** of lungs and blood, and histopathology **(C)** (200×) of lungs were detected at 4, 24, 48, and 72 hpi, respectively. Bacterial burdens are expressed as means ± SEM. “ND” means “not detect” due to mice death.

A hypervirulent *A. baumannii* strain LAC-4 was used to establish a lethal mouse pneumonia model. Mice inoculated with high dose (2 × 10^7^ CFU) of *A. baumannii* all succumbed to infection within 48 hpi, while mice inoculated with low dose (2.5 × 10^6^ and 5 × 10^6^ CFU) survived (Figure 1A), and the bacterial burdens began to decline after 24 hpi and became lower at 72 hpi (Figure 1B). Mice infected with low dose of *A. baumannii* showed transitional infiltration of inflammatory cells in lungs from 24 to 48 hpi, the lung pathology began to recover within 72 hpi (Figure 1C). However, mice infected with high dose of bacteria resulted in severe lung damage and died around 48 hpi. The lungs from dead mice showed consolidation with severe congestion and inflammatory cell infiltration (Figure 1C). These data suggest that low dose of *A. baumannii* infection can induce a transitional pneumonia in which inflammation might contribute to the bacteria clearance. High dose of *A. baumannii* infection results in a strong and uncontrolled inflammation, resulting in a lethal pneumonia.

### Pulmonary Gene Expression Changes in Response to Lethal *A. baumannii* Pneumonia

**Figure 2:**
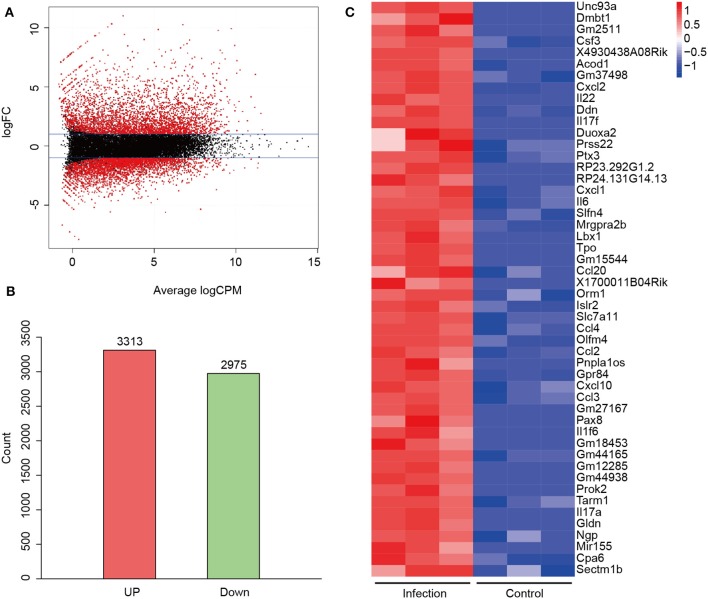
Differentially expressed genes (DEGs) between *A. baumannii*-infected lungs and control lungs. **(A)** MA plot of DEGs. Red dots are DEGs in *A. baumannii*-infected lung with 2-fold change. The black dots reflect no change. **(B)** Gene counts of the upregulated and downregulated DEGs. **(C)** Heatmap of the top 50 upregulated genes sorted by high Log FC. Red means high expression of the genes.

To find out the pathogenesis of *A. baumannii*-induced acute lethal pneumonia, we focused on the innate immune response to *A. baumannii* infection. Rag1^−/−^ mice, which are deficient in T/B cell function, were intratracheally infected with a lethal dose of *A. baumannii*, and the lungs were collected at 24 hpi to perform high-throughput RNA-seq. There were total 6,288 DEGs which had changed over 2-fold and *P*-value < 0.01 in *A. baumannii*-infected lungs compared to the control group (Figure 2A), with 3,313 upregulated genes and 2,975 down upregulated genes (Figure 2B, details in Tables S1, S2). A heatmap of the top 50 upregulated genes sorted by Log FC is shown in Figure 2C, including the genes responsible for pro-inflammatory response (*Il6, Il17a, Il17f, IL-22, Il1f6, Csf3, Ddn, Tarm1*), chemokine (*Cxcl1, Cxcl2, Cxcl10, Ccl2, Ccl3, Ccl4, Ccl20*), transport and metabolism (*Unc93a, Acod1, Slc7a11, Gpr84*). These data indicate that *A. baumannii* lung infection induces strong host inflammatory response at 24 hpi.

### GO Analysis of DEGs of *A. baumannii*-Infected Lungs

**Figure 3:**
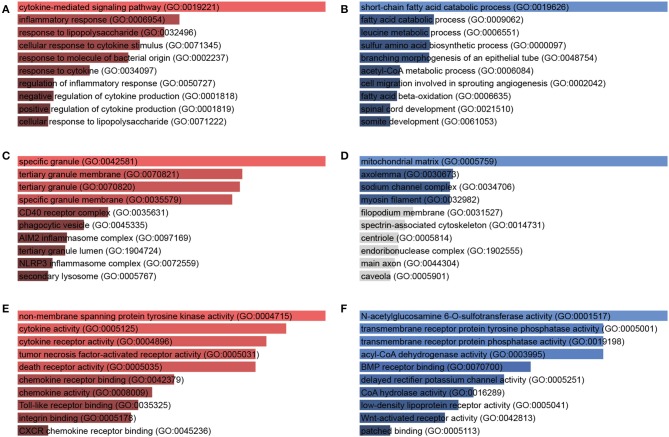
Gene Ontology (GO) enrichment analysis of DEGs in response to *A. baumannii* lung infection. Biological process **(A,B)**, cellular component **(C,D)**, and molecular function **(E,F)** of upregulated (red) and downregulated (blue) DEGs between *A. baumannii*-infected lungs vs. control lungs. The bars are sorted by *P*-value ranking, the length of the bar represents the significance of that specific gene term, the brighter the color, the more significant that term is.

To illustrate the function of the DEGs, GO enrichment analysis was done with biological process, cellular component, and molecular function, respectively. In terms of biological processes, the upregulated DEGs were distributed to inflammatory response, cytokine-mediated signaling pathway, response to bacterial components (Figure 3A, Table S1), while the downregulated genes were distributed to short-chain fatty acid catabolic process, amino acid metabolic process, and acetyl-CoA metabolic process (Figure 3B, Table S2). In the cellular component aspect, the upregulated DEGs belonged to specific granule, phagocytic vesicle, and inflammasome (Figure 3C) and the downregulated genes belonged to mitochondrial matrix, axolemma, and sodium channel complex (Figure 3D). In molecular function, the upregulated DEGs were involved in cytokine and cytokine receptor activity, chemokine and chemokine receptor activity, death receptor activity, Toll-like receptor binding, and non-membrane spanning protein tyrosine kinase activity (Figure 3E). The downregulated DEGs were responsible for transmembrane receptor protein phosphatase activity, acyl-CoA dehydrogenase activity, N-acetylglucosamine 6-O-sulfotransferase activity (Figure 3F).

### KEGG Pathway Annotation of DEGs of *A. baumannii*-Infected Lungs

**Figure 4:**
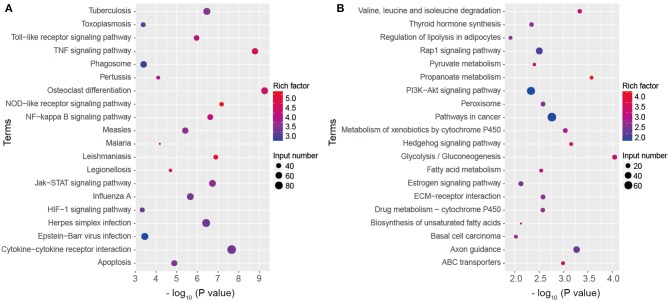
Kyoto Encyclopedia of Genes and Genomes (KEGG) pathway classification of DEGs. Top 20 KEGG terms of upregulated **(A)** and downregulated **(B)** genes in *A. baumannii*-infected lungs at 24 hpi.

To have a deep insight into gene functions of DEGs, KEGG pathway annotation and enrichment analysis were conducted. KEGG analysis showed the significantly upregulated genes were significantly enriched into 55 pathways (*P* < 0.05), including TNF, cytokine-cytokine receptor interaction, Toll-like receptor (TLR), NOD-like receptor (NLR), NF-κB, Jak-STAT, HIF-1 signaling pathways, phagosome, and apoptosis (Figure 4A, Table S1). The downregulated genes were significantly enriched into 35 KEGG pathways, including Valine, leucine and isoleucine degradation, PI3K-Akt signaling pathway, glycolysis/gluconeogenesis, fatty acid metabolism, and ABC transporters, mainly related to nutrient metabolism and biosynthesis, which influenced cell function and survival (Figure 4B, Table S2). The results indicate that multiple inflammatory signaling pathways and metabolic process participate in *A. baumannii*-induced lung injury.

**Figure 5:**
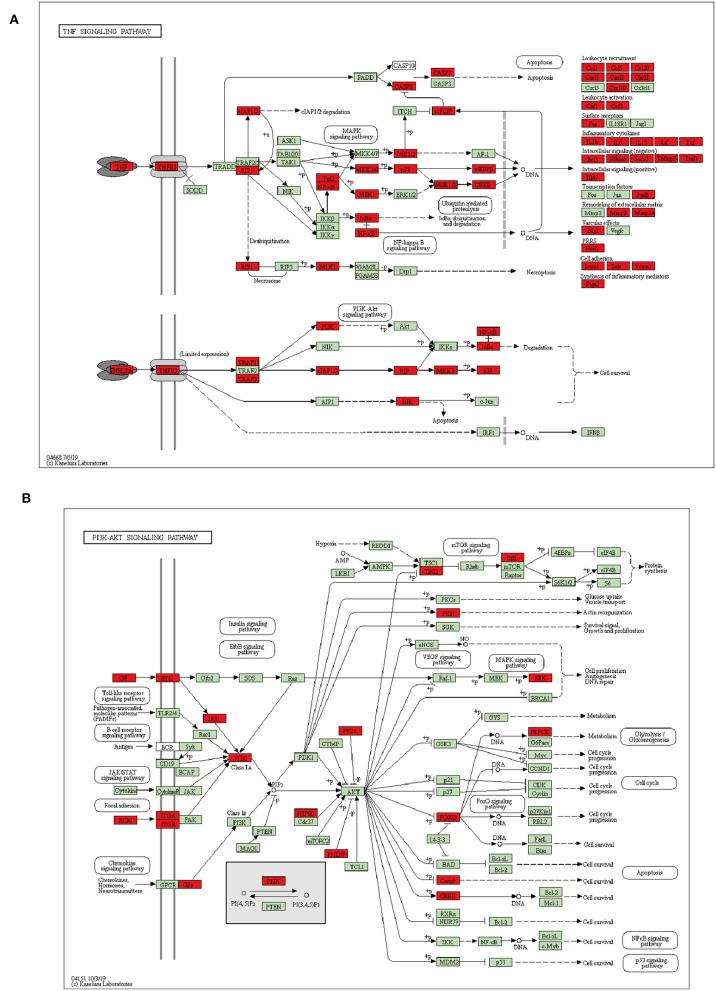
Schematic representation of DEGs enriched in TNF **(A)**, PI3K-AKT **(B)** signaling pathway during *A. baumannii* lung infection. Red color represents DEG, green color represents gene with no difference.

In detail, KEGG showed that 59 of significantly upregulated genes in *A. baumannii*-infected lungs were clustered into the TNF signaling pathway (Figure 5A). These included genes encoding pattern recognition receptors (PRRs), chemokines responsible for leukocyte recruitment (*Ccl2, Ccl5, Cxcl1, Cxcl2, Cxcl3*), inflammatory cytokines *(Il1b, Il6, Il15, Tnf*), genes related to intracellular signaling pathway (*JunB, MAPK, NFkb1, Rela*), extracellular matrix remodeling (*Mmp9 and Mmp14*), positive (*Ifi47*) and negative intracellular signaling (*Bcl3, Nfkbia, Socs3, Tnfaip3, and Traf1*).

KEGG also showed that 99 of significantly upregulated genes in *A. baumannii*- infected lungs were clustered into the cytokine-cytokine receptor interaction, such as the genes encoding cytokines TNF-α, IL-1β, IL-6, IL-12, IL-23, IL-7, IL-15, IL-10, IL-19, IL-22, IL-17a, IFN-γ, IL-3, G-CSF, M-CSF, GM-CSF, which related to survival, proliferation and activation of innate immune cells, chemokines CXCL1, CXCL2, CXCL10, CCL2, which contributed to recruitment of neutrophils and monocytes (Table S1).

The PPRs including TLRs, NLRs, C-type lectin receptors (CLRs), and RIG-I-like receptors (RLRs) are important for pathogen recognition and phagocyte activation. KEGG also revealed that TLR, NLR, RLR signaling pathway, and cytosolic DNA-sensing pathway were enriched in upregulated DEGs (Table S1). For signaling pathway, KEGG enrichment showed that Jak-STAT, NF-kappa B, HIF-1 signaling pathway, and AGE-RAGE signaling pathway in diabetic complications were significantly enriched (Table S1). Also, phagosome pathway and Fc gamma R-mediated phagocytosis, and apoptosis signaling pathway were significantly enriched (Table S1).

For downregulated DEGs in *A. baumannii* infected lungs, PI3K-AKT pathway were significantly enriched, it was associated with cell survival, proliferation, and metabolism (Figure 5B). Many kinds of growth factors such as angiopoietin (*Angpt1*), ephrin (*Efna1, 2, 3*), fibroblast growth factor (*Fgf10, 11*), and vascular endothelial growth factor (*Vegfa, c, d*) were significantly reduced (Table S2). It suggests that cell growth and tissue repair are suppressed after fatal infectious injury of *A. baumannii*.

### Immune Cell Typing of Infected Lung

**Figure 6:**
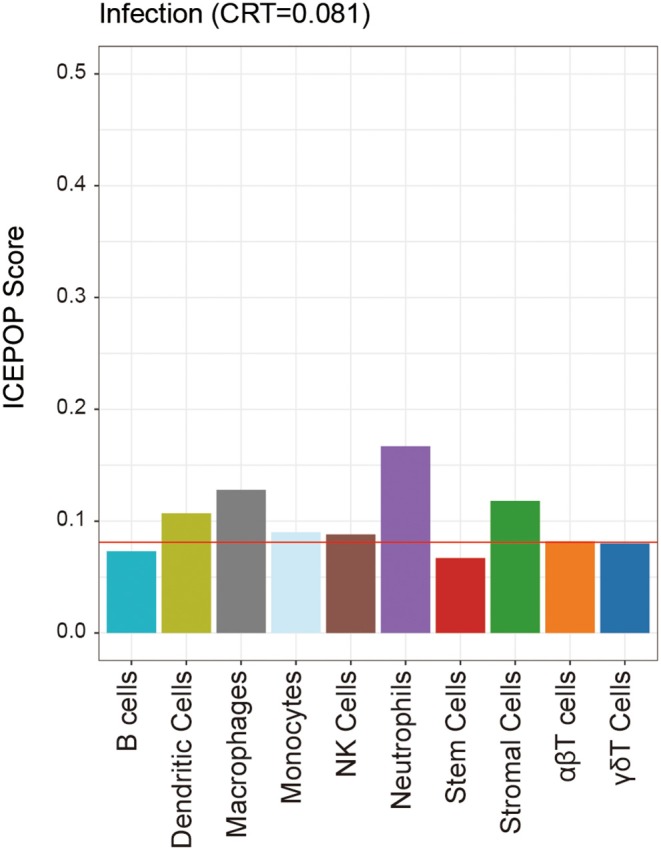
Individual immune cell type responses to *A. baumannii* pulmonary infection. The ICEPOP score (y-axis of the bar graph) represents the relative gene response in each cell type. The cell type response threshold indicated by the red horizontal line represents the threshold value to determine whether a cell type is responding or not.

To explore what kinds of immune cells participated in *A. baumannii* pneumonia, we used ICEPOP to estimate individual immune cell type responses. If a cell type had an ICEPOP score over the cell type response threshold (CRT), the cell type was considered as responsive to the infection. The results showed that neutrophils revealed the highest ICEPOP score among 10 different cell types. In addition, the ICEPOP scores of macrophages, stromal cells, and dendritic cells (DCs) were higher than CRT. The results indicate that these cells may be responsible for host tissue damage after *A. baumannii* pulmonary infection (Figure 6).

### Validation of Selected DEGs by Real-Time PCR

**Figure 7:**
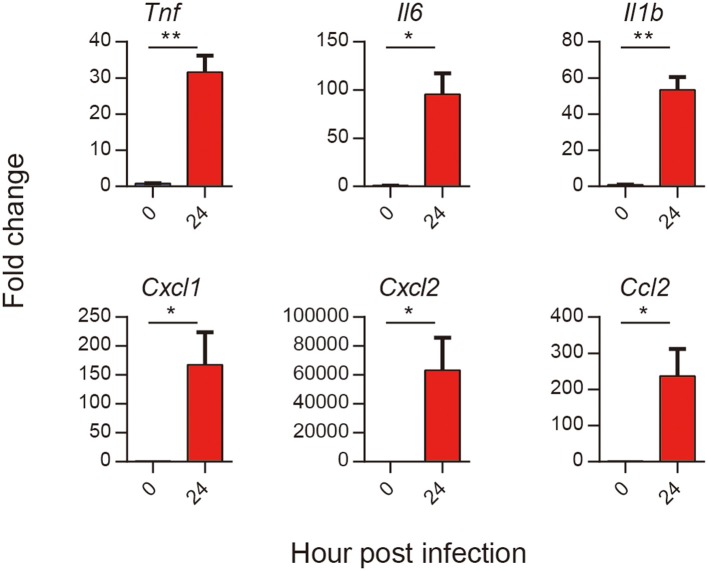
Validation of selected DEGs in response to *A. baumannii* lung infection. C57BL/6 mice were infected with lethal dose (2 × 10^7^ CFU) of *A. baumannii* LAC-4, and lungs mRNA expression of *Tnf*, *Il6, Il1b, Cxcl1, Cxcl2*, and *Ccl2* at 24 hpi were detected by real-time PCR (*n* = 3–5). Bar graphs are expressed as mean ± SEM.*P < 0.05, **P < 0.01.

To test the results of RNA-seq, we selected several proinflammatory cytokines and chemokines to test them. The results showed that all the six selected genes encoding cytokines and chemokines (TNF-α, IL-1β, IL-6, CXCL1, CXCL2, and CCL2) in *A. baumannii* infected lungs were significantly increased at 24 hpi (Figure 7). These were consistent with the expression patterns of six DEGs obtained by RNA-seq, suggesting that the RNA-seq results are reliable to reflect the gene expression trends.

## Discussion

In this study, we established a lethal *A. baumannii* pneumonia model and detected the transcriptome change related to the pathogenesis of lethal pneumonia by RNA-seq. Hypervirulent *A. baumannii* caused an acute lethal pneumonia, supported by severe inflammatory cells infiltration, lung damage and high bacterial burdens. Transcriptome profiling analysis of lung after lethal *A. baumannii* infection showed that a large quantity of DEGs were enriched in multiple inflammation pathways. Immune cell typing emphasized the inflammatory response of innate immune cells headed by neutrophils. The reliability of RNA-seq results were verified with selected DEGs by real-time PCR.

Innate immune cells in lungs are the first line to combat the pathogen. Currently, the early recruitment of neutrophils, monocytes or macrophages is regarded to play a critical role in host resistance to respiratory *A. baumannii* infection, since depletion of neutrophil or macrophage increases the bacterial burdens in lungs (5, 7, 8, 17). In our study, a low dose of hypervirulent *A. baumannii* only caused a transitional lung inflammation without mortality and the bacteria would be cleared at end. In this circumstance, all inflammatory response might contribute to the control of bacteria, which is consistent with the reported protective role of innate immune cells. In our study using a lethal *A. baumannii* infection model, immune cells typing of DEG showed that neutrophils, macrophages, and DCs increased in 24 hpi and the lung pathology also showed severe inflammatory cells infiltration in lungs. These data suggest that extensive infiltration of neutrophils and other inflammatory cells in lung may be responsible for the tissue damage at the late stage during the lethal *A. baumannii* pneumonia. Extensively infiltrated neutrophils will secret proteases, neutrophil elastase, cathepsins, matrix metalloproteinases to combat the bacteria and also cause tissue injury (18). Also, the neutrophil depletion studies demonstrated an important pathogenic role for these cells during *A. baumannii* systemic infection (19). Our previous work showed that neutrophil infiltration with defective bacterial killing function contribute to the severe pneumonia in aged mice (16). The complex role of neutrophil in response to *A. baumannii* infection may be different in non-lethal model vs. lethal model, or in different stages of lethal infection, which needs further study.

Innate immune cells sense the pathogen associated molecular patterns by PRRs and triggers the downstream signaling. The most studied groups of PRRs in the recognition of *A. baumannii* are TLRs and NLRs. Currently, only roles of TLR4, TLR2, and TLR9 are studied (20–22). Our results showed that TLR1, TLR2, TLR3, TLR6, TLR7, TLR9, TLR13 were significantly upregulated in infected mice compared to uninfected mice (Table S1). The roles of other TLRs in *A. baumannii* infection are worthy of further study. Nod1, Nod2, and Rip2 axis are reported to response to *A. baumannii* (23). Consistent with these reports, we also found that numerous genes of NLR signal pathway including *Nod1, Nod2, Nlrp3, Nlrc4, Mefv, Casp1*, and *Casp8* have significantly changed after infection. Nod1 and Nod2 can control intracellular *A. baumannii* through the production of β-defensin 2 in an *in vitro* cell culture models (23). However, using a sub-lethal mouse pneumonia model, Nod2-deficient mice elicit early enhanced production of inflammatory cytokines and chemokines during *A. baumannii* infection, suggesting that Nod2 is required for the early but not the late innate immune clearance of *A. baumannii* pneumonia (24). We also found that the other two groups of PRRs, CLRs and RLRs were also differentially upregulated (Table S1). Their roles in *A. baumannii* infection are not studied yet.

Our RNA-seq data also showed that NF-κB, Jak-STAT, MAPK, signaling activation and subsequent cytokine, chemokine production were upregulated in response to *A. baumannii* infection. These data are consistent with previous study showing that some inflammatory cytokines, such as TNF-α, IL-1β, IL-6, IL-10, IL-17 and chemokines including CXCL1, CXCL2, CCL2, are significantly elevated in LAC-4-infected mice at 24 h post infection (9). It has been recognized that NLRP3 inflammasome/IL-1β signaling mediate lung pathology in *A. baumannii* pneumonia model, since NLRP3 and IL-1 receptor-deficient mice showed reduced pathology (14, 25, 26). Consistent with these results, our RNA-seq data also suggest the critical role of inflammasome pathway, including IL-1β, NLRP3, NLRC4, ASC, caspase-1, and caspase-8 (Table S1). Our results point out the role of IL-17 signaling pathway in the pathogenesis of *A. baumannii* pneumonia. IL-17 is not important for protection in an intraperitoneal *A. baumannii* infection, assessed using antibody neutralization and IL-17A-deficient animals (19), but its role in *A. baumannii* pneumonia remains unknown. In addition to IL-17A, IL-17C, and IL-17F were also elevated in *A. baumannii* pneumonia, their roles need further illustrated. In addition, our results showed many other cytokines such as IL-19 and IL-22, and some inhibitory factors including IL-10, TNFAIP6, ZC3H12A, ACOD1, TNFAIP3, NLRP3, SLAMF1 were also upregulated during *A. baumannii* pneumonia. The balance between pro-inflammatory and anti-inflammatory effects may impact the outcome of infection.

The chemokine receptors CXCR2, CCR1, CCR5, and CCR7 are significantly upregulated. Also, CXCL1, CXCL2, CXCL3, CXCL5, CXCL6 are upregulated, which can bind to CXCR2 on neutrophil to recruit their infiltration. CCR1, CCR5 are mainly expressed on monocyte and significantly upregulation in response to *A. baumannii* infection, indicating the recruitment of monocytes.

Furthermore, our data demonstrated that cell death pathways were significantly activated after lethal *A. baumannii* lung infection (Table S1). KEGG pathway analysis revealed apoptosis related genes included *Casp7, Casp8*, and *Bcl2l1* were upregulated. GO enrichment showed that expression of pyroptosis related genes, such as *Casp1* and *Casp4* significantly increased, as well as necroptosis process related genes *Mlkl* and *Ripk1* (Table S1). These data are consistent with the previous study showing that *A. baumannii* infection induced cell apoptosis, pyroptosis, and necroptosis in BMDM (13, 14), DCs (27), and epithelial cells (28). The key mechanisms in regulating cell death presses in *A. baumannii* lung infection remain to be investigated.

Interestingly, various metabolism pathways were changed in *A. baumannii*-induced pneumonia. Recruitment of neutrophils, macrophages, and DCs requires a lot of ATP to support actin remodeling during infection (29). In comparison to glycolysis, oxidative phosphorylation can apply more ATP, this may be the reason why glycolysis/gluconeogenesis pathway downregulated after *A. baumannii* infection (Figure 4B, Table S2). In addition to ATP production, activated macrophages reprogram TCA cycle intermediates for functions including cytokine production and oxidative burst (29). We found that HIF-1 signaling pathway, the downstream pathway of PI3K-AKT, was upregulated in *A. baumannii* induced pneumonia (Table S1). HIF-1α level shows increase in *A. baumannii* induced mouse sepsis model (30). But the role of HIF-1 signaling pathway in *A. baumannii* lung infection remains unknown. HIF-1α can increase phagocyte intracellular bactericidal function, promotes its granule protease production and release of NO and TNF-α (29). Myeloid specific HIF-1α-deficiency impairs ATP production and inflammatory function (31). Thus, the balance of metabolism pathways is critical for the pro-inflammatory function of innate immune cells during *A. baumannii* pneumonia. Meanwhile, the significantly downregulated genes are clustered into glycolysis, propanoate metabolism, ABC transporters, fatty acid metabolism and so on (Figure 4B, Table S2), indicating the impaired cell function, which reflects the severe tissue damage and correlated with the lethal pneumonia.

It was also interesting to note that *Mir155* was robustly expressed in the top 50 upregulated genes of *A. baumannii*-infected lungs (Figure 2C). Also, 41 microRNA were found upregulated in *A. baumannii* infection, such as *Mir155, Mir147, Mirt2, Mir6972, Mir155hg, Mir7062, Mir6381, Mir6953* were elevated in response to *A. baumannii* infection (Table S1). MicroRNA has been shown to regulate host response against *Salmonella typhimurium* (32). But there are no reports about the role of microRNA in *A. baumannii* infection.

In conclusion, we characterize the global change of transcriptome in an *A. baumannii*-induced lethal pneumonia by RNA-seq. So far, there is no published study on the host transcriptome analysis of *A. baumannii* infection. These data provide an insight into the pathogenesis of the lethal *A. baumannii* lung infection. Our data suggest that strong and uncontrolled inflammatory response results in lung injure. It may be the reason why mice infected with high dose of *A. baumannii* showed severe tissue damage and finally died. However, more experiments are needed to illustrate the role of these pathways in *A. baumannii* pathology. This observation also highlights the need to balance the pro- and anti-inflammatory responses to pathogen such that there is sufficient inflammation to eradicate the pathogen while not severely injuring the host.

## Data Availability Statement

Raw data files for RNA-seq have been deposited in the NCBI Gene Expression Omnibus under accession number GEO: GSE143597.

## Ethics Statement

The animal study was reviewed and approved by Animal Ethical and Experimental Committee of the Amy Medical University.

## Author Contributions

YS and QZ conceived and designed the study. XZ, HG, and LP performed the experiments. XZ, YY, and YS contributed to the data analysis. XZ, NW, YS, and QZ wrote the manuscript.

## Conflict of Interest

The authors declare that the research was conducted in the absence of any commercial or financial relationships that could be construed as a potential conflict of interest.

## Supplementary Material

Table S1 Upregulated DEGs in *A. baumannii* infected lungs and related GO, KEGG pathway lists.

Table S2 Downregulated DEGs in *A. baumannii* infected lungs and related GO, KEGG pathway lists.
